# Magnetically Controlled Transport of Nanoparticles in Solid Tumor Tissues and Porous Media Using a Tumor-on-a-Chip Format

**DOI:** 10.3390/nano14242030

**Published:** 2024-12-17

**Authors:** Tatiana Zimina, Nikita Sitkov, Ksenia Brusina, Viacheslav Fedorov, Natalia Mikhailova, Dmitriy Testov, Kamil Gareev, Konstantin Samochernykh, Stephanie Combs, Maxim Shevtsov

**Affiliations:** 1 Department of Micro and Nanoelectronics, St. Petersburg Electrotechnical University “LETI” (ETU “LETI”), Prof. Popova Str., 5, 197022 St. Petersburg, Russia; tmzimina@gmail.com (T.Z.); kebrusina@gmail.com (K.B.); dtestov@bk.ru (D.T.); 2 Personalized Medicine Centre, Almazov National Medical Research Centre, Akkuratova Str. 2, 197341 St. Petersburg, Russia; fedorovvs.biotech@gmail.com (V.F.); natashashed1@gmail.com (N.M.); kggareev@yandex.ru (K.G.); neurobaby12@gmail.com (K.S.); 3 Department of Radiation Oncology, Technische Universität München (TUM), Klinikum Rechts der Isar, Ismaninger Str. 22, 81675 Munich, Germany; stephanie.combs@tum.de

**Keywords:** tumor, magnetic nanoparticles, magnetically controlled transport, microfluidic systems, organic porous systems, tissue engineering

## Abstract

This study addresses issues in developing spatially controlled magnetic fields for particle guidance, synthesizing biocompatible and chemically stable MNPs and enhancing their specificity to pathological cells through chemical modifications, developing personalized adjustments, and highlighting the potential of tumor-on-a-chip systems, which can simulate tissue environments and assess drug efficacy and dosage in a controlled setting. The research focused on two MNP types, uncoated magnetite nanoparticles (mMNPs) and carboxymethyl dextran coated superparamagnetic nanoparticles (CD-SPIONs), and evaluated their transport properties in microfluidic systems and porous media. The original uncoated mMNPs of bimodal size distribution and the narrow size distribution of the fractions (23 nm and 106 nm by radii) were demonstrated to agglomerate in magnetically driven microfluidic flow, forming a stable stationary web consisting of magnetic fibers within 30 min. CD-SPIONs were demonstrated to migrate in agar gel with the mean pore size equal to or slightly higher than the particle size. The migration velocity was inversely proportional to the size of particles. No compression of the gel was observed under the magnetic field gradient of 40 T/m. In the brain tissue, particles of sizes 220, 350, 820 nm were not penetrating the tissue, while the compression of tissue was observed. The particles of 95 nm size penetrated the tissue at the edge of the sample, and no compression was observed. For all particles, movement through capillary vessels was observed.

## 1. Introduction

The development of methods for the selection of efficient and low-toxicity drugs for target delivery and the suppression of tumors growth locally, thus minimizing damage to healthy organs, is an urgent and acute biomedical problem [1,2,3,4,5]. An intensively developing approach is the utilization of magnetic nanoparticles (MNPs) as vehicles for the targeted delivery of anticancer drugs. The realization of this approach requires solving several problems: the development of external fields providing the spatially determined transport of particles within an organism, the preparation of biocompatible and chemically stable MNPs and their chemical modification with targeting components (e.g., antibodies, peptides, etc.), ensuring their presence at the surface of pathological cells, and releasing antitumor preparations at the target cells [5,6,7,8,9].

References to magnetic nanoparticles (MNPs) for biomedical applications have been around for quite some time. Thus, in [10], the authors discuss applications of magnetic materials in medicine, including applications of the technology of fine particle iron for the treatment of cancer and mental disease, and for diagnostic purposes. Although the real explosion of nanoparticle technologies for biomedicine occurred with the introduction of nanotechnology [11], various applications of MNPs started to intensively develop, particularly in the field of targeted drug delivery [5,12,13,14,15,16]. The most recent efforts are concentrated on the development of various types of coatings for magnetic nanoparticle core [5,17,18,19,20,21,22]. So, at present, the family of biomedical magnetic nanoparticles includes the following principal types: maghemite (γ-Fe_2_O_3_) [23] and magnetite (Fe_3_O_4_) [11], used as a magnetic core, SPIONs [6,7,9,24], magnetic liposomes–MNPs covered with a phospholipid layer [25], and others. Among the biogenic magnetic nanoparticles, magnetosomes should be mentioned, as they are generated by magnetotactic bacteria. These MNPs demonstrate a magnetic core with an outstanding ordered structure and are covered with a layer like a cell wall [5]. It is necessary to note that the isolation of such MNPs is a very difficult and labor-intensive process [26,27,28].

Contemporary studies in the field of MNPs, particularly in the most current field of development, target-oriented drug delivery particles, are focused on the following directions [5]: the regular structure of MNPs, the biocompatibility and chemical neutrality of the shell, and, at the same time, the possibility of its chemical modification [29], the absence of particle aggregation effects, small size, and the ability to penetrate living tissues.

Solving the above problems at the stage of pharmaceutical preparation development, as well as at the stage of dosage adjustment in personalized medicine and target delivery challenges, is labor-intensive, expensive, and requires the use of laboratory animals, volunteers, etc. A promising solution is the preparation of tumor models on a chip [30,31,32,33,34,35,36,37]. This could be used in extensive experiments on drugs efficiency adjustments, as well as with the samples of patients’ tumor cell culture to adjust personal biochemistry and the dosage of the antitumor drug.

The ultimate goals of solving these problems involve creating a topology of microchannels that imitate the vascular system, compartments for the culturing of tumor cells with interfaces with the vascular system and that allow access for tested drugs, special cells for studying the migration of drugs into the culture of tumor cells, and models based on polymer gels similar to living tissues.

The current technological level of hybrid-integrated microfluidic analytical devices fabrication allows the implementation of a wide variety of topologies, thick layer or printing technologies, sheet and nanostructured materials, integrated sensors. and many other possibilities. The precision of realization approaches 1–5 μm [38,39,40,41]. However, the integration of live tissues, including 2D, 3D, or spheroid types, represents a specific challenge [38].

A variety of approaches using models of tumors have been described [5] the advances which have been realized within the tumor-on-a-chip (TuOCh) concept design. But despite their advantages, such as simple culture growth, low cost, and the technological availability of formation and assembly methods, these approaches involve subjecting cancer cells to artificially restricted growth conditions and lack key components of the tumor microenvironment that affect cancer biology and drug response, including tumor mechanical properties and intertumoral gradients of compounds. In healthy organs, the vasculature is solid, while in tumors, the vasculature is leaky, with poor lymphatic drainage [36]. Nanoparticles of <200 nm in size are reported to be selectively captured in a tumor by an effect known as enhanced permeability and retention (EPR) [36,37]. The EPR effect in human tumors is of great interest, since it opens a way of drug delivery when the permeability of tissues is normally poor. Thus, the study of tumor models on a chip can help to find ways to increase the permeability of nanoparticles delivering antitumor drugs to targets by using vasculature [42], the EPR effect, or some other mechanisms for the targeted delivery of drugs in nanomedicine. The effect of magnetically guided target delivery was demonstrated in [43] for dextran-coated MNPs of 50 nm size on a murine model. Recently, a dual-mode targeting implementing EPR and ligand–antibody interaction have shown great prospects for the in vitro use of magnetic nanoparticles in therapy. As an example, single core SPIONs carrying siRNA and HER2 antibody fragments have demonstrated high biocompatibility and remained stable and resistant to enzymatic degradation in the models of biological fluids. These particles demonstrated improved intake and retention on HER2-overexpressing breast cancer cells compared to onloaded particles even in the magnetic field. Similarly, <100 nm size core SPIONs functionalized with RGD and glucosamine, aside from biocompatibility, expressed low macrophage activation. Utilizing EPR and ligand specificity, the authors confirmed high SPION retention and imaging effectiveness in tumor tissue.

At the current stage of work, the aim was to study the behavior of a variety of magnetic nanoparticles, including the “bare” magnetite nanoparticles (mMNPs) and carboxymethyl dextran-coated SPIONs (CD-SPIONs) as regards the penetrability into various porous tissues, including synthetic gels and murine brain tissue under the magnetic field drive. The results are important for the development of tumor-on-a-chip models and the target-oriented transport of anticancer medicines in organisms.

## 2. Materials and Methods

### 2.1. Magnetic Nanoparticles

Two types of magnetic nanoparticles (MNPs) were synthesized and studied, namely (1) MNPs without a shell (mMNPs) [44,45,46,47] and (2) carboxymethyl dextran-coated superparamagnetic iron oxide nanoparticles (CD-SPIONs) [48,49,50,51]. The MNPs were synthesized by authors as described below.

#### 2.1.1. Materials for Preparation of Magnetite Nanoparticles

The following reagents were used for the preparation of mMNPs: ferric chloride hexahydrate FeCl_3_·6H_2_O, ammonium hydroxide, NH_3_OH and sodium tetrahydridoborate, NaBH_4_—all are of puriss grade, ≥98%, obtained from LLC “Vecton” (St. Petersburg, Russia).

#### 2.1.2. A Procedure of Magnetite Nanoparticles Formation

The mMNPs were synthesized by the proprietary method based on the reduction principle at ETU "LETI" (St. Petersburg, Russia).

The 1.25% *w*/*v* solution of FeCl_3_·6H_2_O was prepared in deionized water under intensive vertical stirring. The 3.5% *w*/*v* solution of NH_3_OH was prepared in deionized water. Then, 2 g of NaBH_4_ was dissolved in 100 mL of 3.5% solution of NH_3_OH. The solution of FeCl_3_·6H_2_O heated up to T = 40 °C and the solution of NaBH_4_+NH_3_OH was added to it under intensive stirring. Then, the system was heated to achieve thermodynamic equilibrium at T = 95 °C. Under these conditions, the reduction reaction was completed in 60–120 min. The magnetic nanoparticles were formed during the reduction process, being settled at the bottom of the vessel under the action of a magnetic field. In two hours, the system cooled down to room temperature, and the magnetic phase was isolated using the magnetic separation technique. As a result, a highly stable, black magnetic powder in the form of two fractions of Fe_3_O_4_ was settled at the bottom of the reaction vessel.

#### 2.1.3. Materials for Preparation of Carboxymethyl Dextran-Coated Superparamagnetic Iron Oxide Particles (CD-SPIONs)

The reagents used—FeSO_4_ and FeCl_3_ aqueous solutions, NH_4_OH, CsCl, and carboxymethyl dextran sodium salt—all are of puriss grade and were obtained from Sigma-Aldrich (St. Louis, MO, USA).

#### 2.1.4. Procedure of CD-SPIONs Preparation

Carboxymethyl dextran-coated superparamagnetic iron oxide nanoparticles (CD -SPIONs) were synthesized by the microemulsion method at Institute of Cytology RAS as described earlier [52,53] and more specifically by the coprecipitation of Fe^2+^ and Fe^3+^ salts solutions (at pH = 10, inert atmosphere and T = 80 °C) and followed by electrosteric stabilization by a negatively charged dextran coating. The resulting core of CD-SPIONs are 30 nm size.

### 2.2. Microfluidic Chips

#### 2.2.1. Materials for Microfluidic Chips Fabrication

The layers of microfluidic chip sandwich structures were prepared by laser ablation from a poly(methylmethacrylate) (PMMA) sheet, 1.5 mm thick, polyethylene terephthalate (PET) sheet 0.5 mm thick, polyethylene (PE) sheet 0.3 mm thick, polypropylene (PP) sheet (Astroplastica, St. Petersburg, Russia). All layers are optically transparent in the visible range of the spectrum and sufficiently chemically and biologically inert (Table 1). In certain cases, 3M™ Scotch^®^ Transparent Film Tape 600 (St. Paul, MN, USA) (polyvinylchloride (PVC) film/acrylic adhesive) was used for hermitization [39,40] (Table 1).

#### 2.2.2. Microfluidic Chips Fabrication Technology

The microfluidic chips were fabricated as a sandwich structure bonded by the thermo-compression technique [39,63,64,65,66]. The surfaces were treated by isopropyl alcohol prior to bonding procedure. The temperature of bonding was 120 °C, which was applied under gradual heating stepwise with the intervals of 20 °C and exposition time at each step of 20 min. The pressure was about 200 g/cm^2^.

The topologies of planar capillary patterns were formed in polymer sheets of 0.3–0.5 mm thickness using the laser ablation technique with a laser machine Zareff M2, 300 × 200 mm, 40 W CO_2_ laser (LLC “Zareff”, Moscow, Russia). The sandwich structures were composed by adjusting the base layer and cover layer (with corresponding openings) with the intermediate layer comprising through in-sheet channel structures. The assembly was placed into the steel press (200 g/cm^2^) and heated in oven Industrial Lab DZG-6020 25L Electro-Thermostatic Hot Air Circulating Drying Oven (Zhejiang NADE Scientific Instruments Co. Ltd., Hangzhou, China) at 120 °C for 60 min.

### 2.3. Porous Media of Differing Mean Pore Diameter

#### 2.3.1. Materials for Preparation of Porous Media

Porous media as a model of biological tissues in experiments on magnetically mediated transport of nanoparticles were prepared using agarose gel (Agar-Agar 900, LLC MolecularMeal, Moscow, Russia) of differing concentrations of initial suspension.

#### 2.3.2. Procedure of Porous Media Preparation

The mean pore size concentration dependence was selected according to the data presented in earlier publications [67,68]. The mean pore size of the gel varied by the selection of polymer concentration.

To prepare agarose gels, the powders were suspended in water of ambient temperature and left to swell for 20 min. After swelling, the suspended polymer was slowly heated under continuous stirring up to 90 °C for 30 min. Then, the gel was poured into cells of the hybrid microsystem using a dispenser. Agar gels were prepared at various concentrations within the range from 0.25% *w*/*v* to 0.5% *w*/*v*. The gel models were optically sufficiently transparent.

### 2.4. Testing Materials, Buffer Solutions

Phosphate buffer solution was prepared from PBS tablets (Biotechnology grade, Am-E404-100, Helicon, Moscow, Russia) composed of 0.01 M PBS, 0.137 M NaCl, 0.002 M KCl, and pH 7.4 dissolved in 100 ml of deionized water. Albumin bovine serum (BSA) and heat shock isolation (Biotechnology grade. Amresco Inc., Framingham, MA, USA) were used in preparation of the blood serum model.

### 2.5. Methods

#### 2.5.1. Collection of Laboratory Mouse Brain Tissue Sample

The mouse was humanely sacrificed by severing the brainstem under anesthesia. Anesthesia was induced with propofol; then, the mouse was placed in a chamber where the inhalation of anesthesia with sevoflurane was performed [69]. Under sterile conditions, a linear incision of soft tissues in the projection of the sagittal suture was made. The parietal, frontal and occipital bones were skeletonized, and a craniotomy was made using a diamond bur. Then, the brainstem was transected with a scalpel, a frontal slice of the right hemisphere of the brain was taken in the parietal lobe area, and the brain tissue material was placed in a container filled with saline.

The animals (weighing 250–300 g) were purchased in an animal nursery “Rappolovo” RAMN (St. Petersburg, Russia). All animals were bred and kept under specific pathogen-free conditions in accordance with the guidelines of the Federation of European Laboratory Science Association (FELASA). All animal experiments have been approved by the Ethics Committee of the Almazov National Medical Research Centre of the Ministry of Health of the Russian Federation (Protocol number 05112019 dated 11 November 2019) and were in accordance with institutional guidelines for the welfare of animals.

#### 2.5.2. Application of Magnetic Field in the Microfluidic System

**Figure 1:**
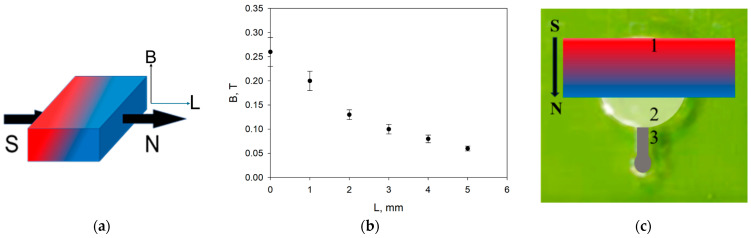
Geometry of Nd-Fe-B magnet of 30 mm × 10 mm × 4 mm size strength of magnetic field (MF) at the surface value of 0.27 T (**a**); MF gradient versus sample length of ~0.04 T/mm (**b**); positioning of magnet at the chip with gel and suspension of nanoparticles compartment (**c**); 1—neodymium magnet, 2—agar gel, *R* = 5 mm, *h* = 0.3 mm, 3—aqueous suspension of MNPs.

The neodymium magnet (class N35; with a nickel protective coating) with a magnetic field (MF) strength at the surface of 0.27 T (Figure 1a) and magnetic field gradient in the near field of 0.04 T/mm (Figure 1b) was used, which, according to the literature data [70], corresponds to the MF forces of magnets used in medicine. The form applied in the experiments’ magnetic field strength distribution, i.e., the relationship of the magnetic field strength versus distance along the chip channel from the magnet, is illustrated in Figure 1b, and the arrangement of the magnet at the top of the microchip compartment with the gel is presented in Figure 1c.

## 3. Results

### 3.1. Features of Manufacturing Microfluidic Systems

**Figure 2:**
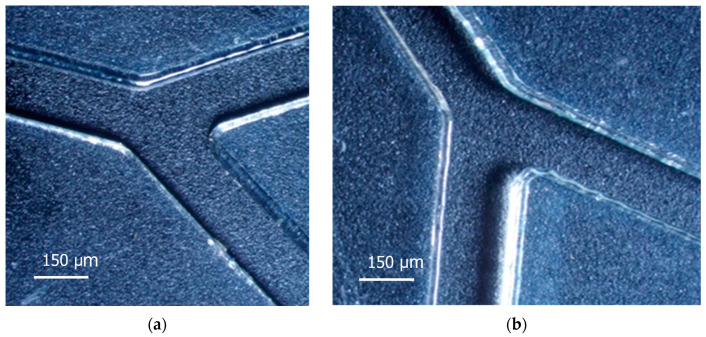
Microscopic image of channels 150 μm wide and 300 μm deeply fabricated using the laser ablation technique at different laser head speed values: 10 mm/s (**a**) and 15 mm/s (**b**).

The channels of microfluidic systems (MFS) were prepared by laser ablation. Their quality depends on the velocity of the laser head movement and the laser power. As presented in Figure 2a, the velocity of 10 mm/s gives better results, as regards the quality of the cut edge, than the ablation at 15 mm/s (Figure 2b). In the second case, the wall of the channel is ribbing, and the border has ridges and sagging, which scatter light.

The power of the laser beam in both cases was 8 W. At lower power, it was not possible to achieve a through-cut ablation in the material 0.3 mm thick, while at higher speed, the cracking of PMMA was observed along the edges due to the occurrence of mechanical stresses around high-temperature gradients in polymer material.

**Figure 3:**
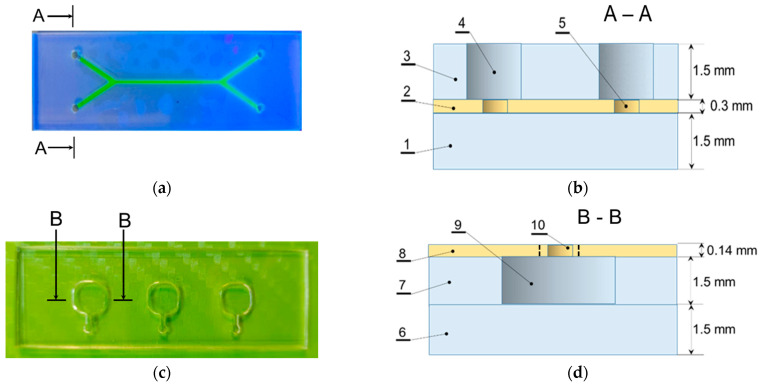
Photographs of cross-sections of microfluidic chips. (**a**)—a schematic of type assembly and (**b**) photographs of MFS-1, where 1—base layer, 2—microfluidic layer, 3—casing layer, 4—inlet, 5—microfluidic channel and MFS-2 (**c**,**d**) for MNP analysis in liquid media; (**c**)—a schematic of type assembly and photograph (**d**) of MFS-2 for tissue-on-a-chip type studies, where 6—base layer, 7—layer for tissue sample, 8—casing layer, 9—tissue sample cell, 10—inlet.

The assembly of the multilayer polymer microfluidic systems was performed via thermocompression bonding of the thermoplastic polymers, including PMMA, PET, PE and PP (Table 1). The thermocompression technique makes it possible not only to seal the layers of the sandwich-like system but also to form certain 3D elements, such as inlet basins, lenses etc. within the same technological cycle [71,72,73]. In this work, two types of three-layer polymer assemblies of microfluidic systems have been developed: (1) PMMA-PET-PMMA with capillaries for analyzing nanoparticles in liquid media made from the 0.3 mm thick PET functional layer (Figure 3a,b), and (2) PMMA-PMMA-PET with basins for gels (Figure 3c,d). Sealing of the second type of microfluidic systems was carried out using a transparent PET film 140 µm thick with a deposited glue layer.

### 3.2. Features of Magnetic Nanoparticles Migrating in Aqueous Media Patterns

Two types of MNPs were investigated: (1) magnetite (Fe_3_O_4_) nanoparticles without a shell, mMNPs, aqueous suspension of 2 mg/mL concentration and (2) MNPs of iron oxide (Fe_3_O_4_) in carboxymethyl-dextran shell, CD-SPIONs, aqueous suspension of 2.2–4 mg/mL concentration (Table 2).

**Figure 4:**
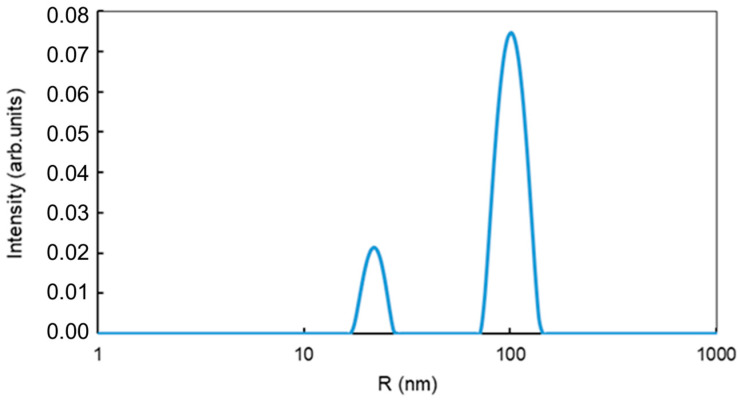
Size distribution of mMNPs obtained by dynamic light scattering technique.

The hydrodynamic radii (*R_H_*) of fractions of mMNPs in the sample were estimated by the laser light scattering using a Photocor Mini (Photocor LLC, Moscow, Russia) as 23 nm and 106 nm, (Figure 4). The concentration of MNPs in suspension is 2 mg/mL.

**Figure 5:**
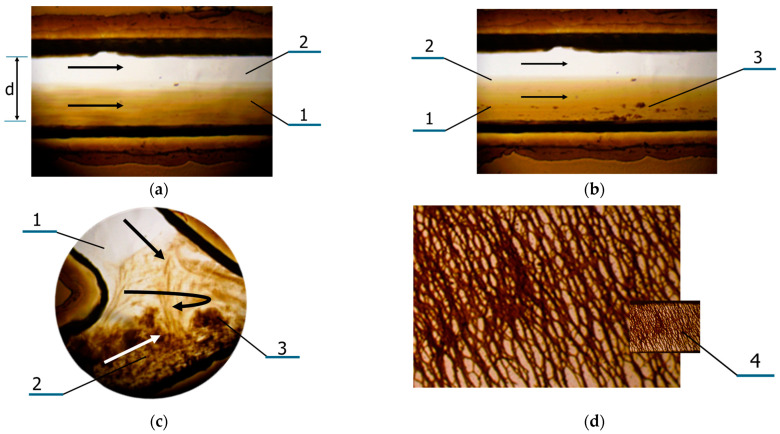
Peculiarities of microfluidic flow for mixture of magnetite nanoparticles (mMNPs) of 23 nm and 106 nm size: (**a**) parallel flow at *V* = 1 mm/s of mMNPs aqueous suspension of 2 mg/mL concentration (1), and water (2) in MFS-1 under MF gradient of 40 T/m in capillary channel 200 μm wide (**d**) and 300 μm deep at an initial time (**a**); in 5 min, when considerable agglomeration (3) is observed (**b**); in 10 min when laminar vortices of mMNP suspension are observed (**c**) and in 20 min when the total grid of magnetic fibers (4) is observed (presented also accelerated image) (**d**).

The experiments on the mobility of the mMNPs in aqueous suspension in capillaries demonstrated intensive agglomeration phenomena, which was also observed earlier elsewhere [40,54,55]. Figure 5a,b show parallel flows of a suspension of mMNPs and aqueous medium in MFS-1 at the initial time (Figure 5a) and 5 min after the start of the experiment (Figure 5b), when the formation of agglomerates of an approximate size range of 0.5–2.0 μm was observed within several minutes. When BSA solution of 7% *w*/*v* was used as the suspension medium, agglomeration was accelerated. Further development of the process led to the formation of agglomerates and appearance of laminar vortices (Figure 5c). After some time of exposition to a static magnetic field (MF) gradient of 40 T/m, the formation of spatial grid-like structures of the type shown in Figure 5d were observed. These intensive agglomeration effects obviously make it impossible to study the diffusion of this type of MNPs and use them as delivery particles in organisms.

CD-SPIONs were synthesized by the microemulsion method at Institute of Cytology RAS as described earlier [23,24,25,26] and more specifically by coprecipitation from Fe^2+^ and Fe^3+^ salt solutions (pH = 10, t = 80 °C) and stabilization by a cross-linked dextran coating. The hydrodynamic size and zeta potential of CD-SPIONs were assessed by dynamic light scattering (DLS) using a Zetasizer Nano (Malvern Instruments, Malvern, UK). The concentration of iron in the nanosuspension was determined by the thiocyanate method as 0.2% *w*/*v* [6].

**Figure 6:**
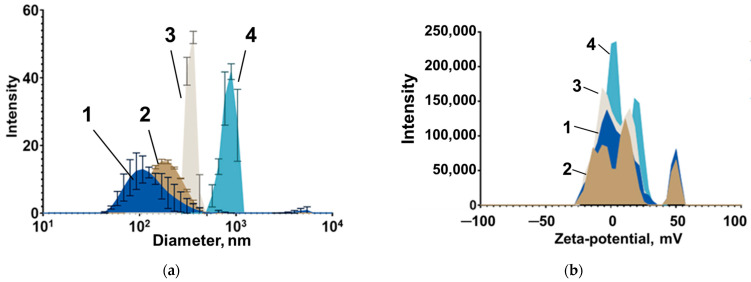
Hydrodynamic diameter (**a**) and zeta-potential (**b**) distribution for CD-SPIONs of various diameters (1—95 nm, 2—220 nm, 3—350 nm, 4—820 nm).

DLS results for the sizes of CD-SPIONs are shown in Figure 6a. The investigated nanoparticles were sufficiently monodisperse in size with the main distribution peak corresponding to the expected nanocluster diameter. The major peak for the surface charge of all samples was approximately 0 mV, since non-functionalized dextran lack charged sites (Figure 6b). Lower monodispersity, especially for the 820 nm sample, can be explained by the uneven polymer distribution on the surface of the Fe core. TEM images of CD-SPIONs of different sizes are given in the Supplementary Materials.

The resulting suspensions have a high [Fe^3+^, Fe^2+^] concentration of ~4 mg/mL and contain superparamagnetic (as was evident from their susceptibility for external magnetic field) particles with dominating fractions of mean size 95 nm, 220 nm, 350 nm and 820 nm (highly heterogeneous), as presented in Figure 6. The concentration of iron in the nanosuspensions was determined by the thiocyanate method as described in [6]. The CD-SPIONs are described in Table 2.

### 3.3. Study of Magnetically Controlled Migration of SPIONs in Porous Media Modeling Life Tissues

The magnetic field makes it possible to deliver MNPs via blood vessels to the tumor and concentrate MNPs near its surface. The localization of MNPs near the tumor surface greatly simplifies the delivery of drugs into the affected tissues. The migration of MNPs in tissues is a rather slow process, the rate of which depends on the parameters of the tissue, such as density and pore size, on the characteristics of MNPs, their size, shape, shell type, magnetic moment of MNPs and on the strength of the magnetic field (MF) [71]. In this work, the migration of CD-SPIONs of different sizes in the porous media of agar gel samples of various pore sizes was studied.

**Figure 7:**
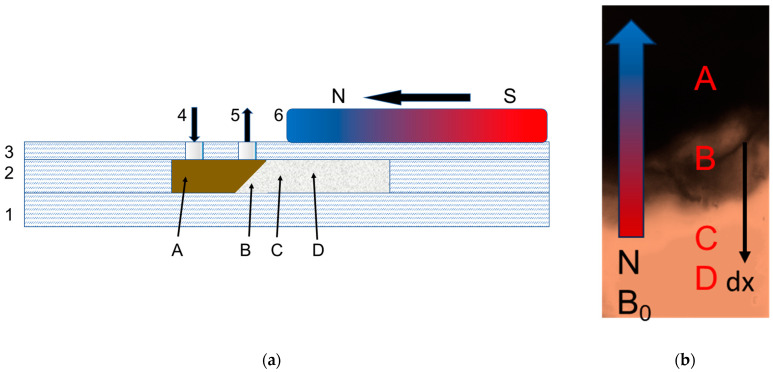
Experimental setup of microchamber (**a**) for magnetically controlled migration study and the position of the magnet above the sample (**b**): 1—base, prepared of PMMA layer, 1 mm thick; 2—layer with microfluidic system profile, PMMA, 1 mm thick; 3—PP layer, 0.3 mm thick; 4—inlet for CD-SPION suspension; 5—outlet of CD-SPIONs suspension; 6—neodymium magnet. A—MNP suspension zone, B—interface zone, slope of gel massive, C—zone accessible for CD-SPIONs, D—gel.

Three types of agar gel were selected as defined by the concentration of the dry agar: 0.5 *w*/*v*%, 0.4 *w*/*v*% and 0,25 *w*/*v*%, which corresponds to the pore sizes of 100–150 nm (G1), 200–300 nm (G2), and 350–400 nm (G3) [72,73]. In the agar gels G1 and G2 with the mean pore sizes of 100–150 nm and 200–300 nm, correspondingly, no migration of CD-SPIONs of the sizes 95 nm, 220 nm, 350 nm and 820 nm was observed. The cross-section of the experimental chip is presented in Figure 7.

**Figure 8:**
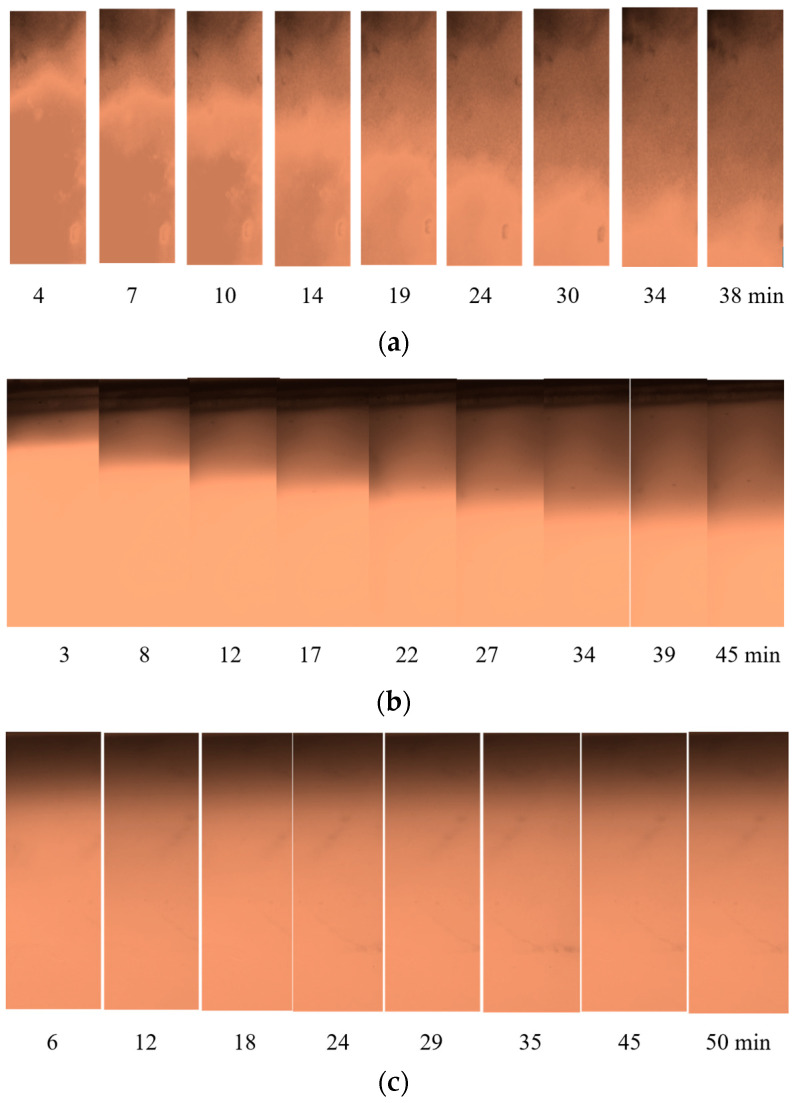
Magnetically controlled fronts of CD-SPIONs (*r* = 95 nm (**a**), 220 nm (**b**), 350 nm (**c**)) in agar gel of 350–400 nm mean pore size, under 40 T/m gradient of MF in microfluidic chip.

In the gel G3, the following patterns were detected (Figure 8).

**Figure 9:**
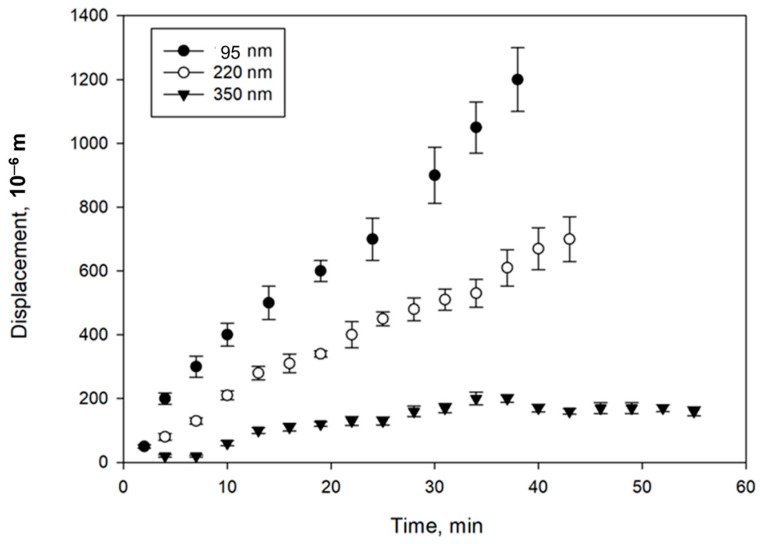
Relationships of magnetically driven displacement of CD-SPIONs of differing size versus time in agar porous medium with the mean pore size of 350–400 nm.

The relationships of magnetically controlled transport of CD-SPIONs in agar gel versus time are presented in Figure 9.

**Figure 10:**
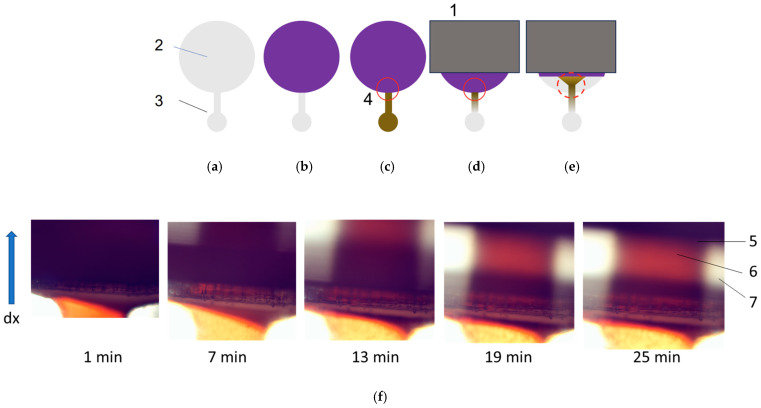
The experimental setup for CD-SPIONs transport in rat brain. (**a**) Geometry of the microfluidic system; (**b**) sample of the rat brain shown in blue; (**c**) CD-SPIONs suspension shown in brown; (**d**) application of magnetic field and migration of CD-SPIONs; (**e**) displacement of the brain tissue; (**f**) photographs of the stages (**c**–**e**) with CD-SPIONs of 350 nm size. 1—neodymium magnet, 2—basin for brain tissue, 3—basin and channel for CD-SPIONs suspension, 4—aperture of observation in the microscope marked with red line, 5—border of the displaced tissue with CD-SPIONs suspension underneath, 6—flow of remaining CD-SPIONs moving in MF, as in (**e**), 7—part of basin for tissue after (4).

The migration characteristics of CD-SPIONs in brain tissue were studied using particles of 350, 220 and 95 nm hydrodynamic size. The schematic of zones monitoring are presented in Figure 10a–e. The brain tissue was sliced and placed into the large basin of MFS-3 with adjustment of the sample height with the depth of the basin. The CD-SPIONs suspension was delivered into the microfluidic channel and small basin as shown in Figure 10c. The pattern developed under the action of the magnetic field was observed via a microscope. The CD-SPIONs of 350 nm mean diameter on the tissue were displaced under the Lorentz force for 2 min (Figure 10e,f).

**Figure 11:**
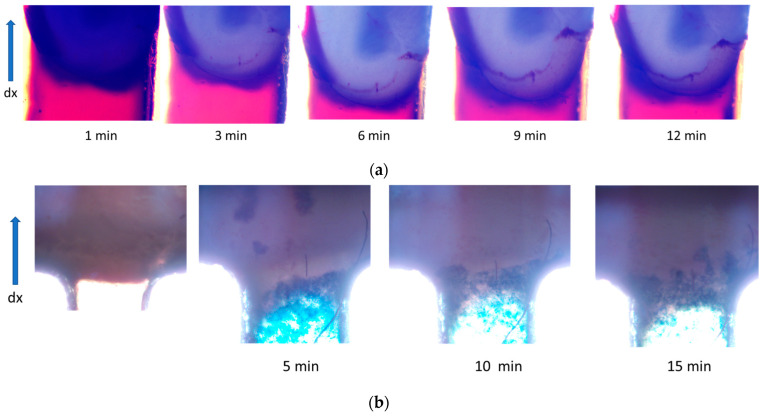
Magnetic transport of CD-SPIONs of the hydrodynamic size 220 nm (**a**) and 95 nm (**b**). Time of the pictures capture was shown below each photograph. In (**b**), the left image shows the brain sample border.

The experiments were completed for the particles of the 220 nm and 95 nm hydrodynamic size. The results are presented in Figure 11.

Figure 11a demonstrates that CD-SPIONs of the size 220 nm could not penetrate rat brain tissue, and at the same time, they are not capable of compressing the tissue. Figure 11a shows the initial stage of the experiment, when the CD-SPIONs suspension is filling compartment 3, while migrating in the magnetic field, positioned vertically in the picture. Phase (b) demonstrates the microchip in a 24 min period, when all the particles moved through the vasculature and slit along the walls of the basin, while the tissue remained intact. The conclusion was checked by the buffer purging the basin after the experiment when all the suspension was washed away.

The experiments with particles of 95 nm hydrodynamic size demonstrated similar patterns. Thus, Figure 11b shows the initial stage, when the CD-SPIONs sample was concentrated near the “tissue–solvent” interface, and the next frame shows the CD-SPIONs penetrating vasculature and the slits between the tissue and the casing.

## 4. Discussion

The study of the transport behavior of MNPs in porous media, including polymer gel (agar) of various mean pore size, as well the live brain tissue, makes it possible to evaluate the character of localization of nanoparticles under the action of a magnetic field. A significant increase in the concentration of MNPs near the surface of the tissue was observed: in several minutes, the concentration of CD-SPIONs increased by 5 to 10 times, depending on the particle size. Moreover, the active movement of CD-SPIONS was observed immediately after the MF was turned on. At the same time, the penetration of CD-SPIONs of hydrodynamic size down to 95 nm into the brain tissue was not observed. The SPIONs suspension penetrates the tissue via the vascular network and the slits near the walls of the microsystem. This means that the lower size particles are needed, which are at a higher price and less available. In this respect, the bacterial magnetosomes could be of interest; however, such materials are also not easily available.

**Figure 12:**
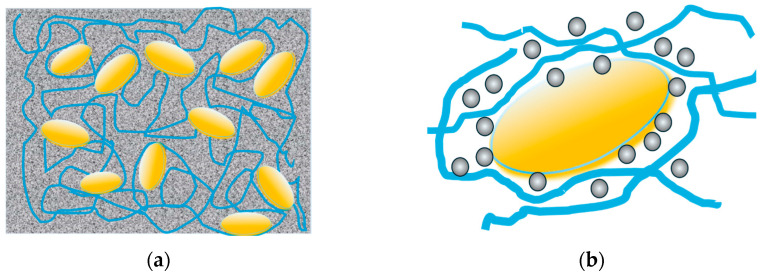
Oncotheranostic system, containing polymer gel of specific pore size (blue lines), fixing the cancer cells and infiltrated with suspension of MNPs (**a**) carrying the selected anticancer drug (**b**).

At the same time, as regards the use of low cost and easily available CD-SPIONs, they could be of good use in express diagnostics systems (oncotheranostics) comprising hybrid materials, including polymer gels that are accessible for MNPs and captured cancer cells [39], as presented in Figure 12. In this case, the express testing of the fixed cells could be made by the targeted delivery of MNPs loaded with selected medications in the tumor-on-a-chip format microfluidic systems [41].

Another direction of MNPs target delivery development approaches is the angiogenesis methods, which will enable delivering drugs using larger size MNPs, which are more available, less expensive and more susceptible to magnetic guidance.

Microfluidic systems developed for modeling tumor tissues (tumor-on-a-chip) are an important tool for studying the transport characteristics of nanoparticles. These systems allow simulating physiological conditions in tumor tissues and vessels, which is especially important when testing new methods for drug delivery. In this paper, we describe systems based on polymer sandwich structures allowing the control and study of nanoparticles transport in capillary channels simulating the vascular system. The experimental results showed that when using a magnetic field, it is possible to achieve the significant localization of nanoparticles on the surface of tumor tissues, which facilitates their delivery to target cells. However, it should be noted that in real tumor tissues, the vascular system differs from normal tissues in its increased permeability, which requires careful calibration of the magnetic field strength and nanoparticle sizes.

To model tissues in the experiments, porous media based on agar gels with different mean pore sizes were used. These models allow the tissue characteristics such as density and porosity to be reproduced, which makes it possible to evaluate the transport characteristics of nanoparticles. In addition, such environments are easily integrated into microfluidic systems, which allows us to create complex research systems on a chip with their help.

Based on the obtained data on the transport of MNPs in tumor tissues, several promising areas for future research can be identified. These areas may focus on improving nanoparticle delivery methods, the in-depth study of biological interactions, and the optimization of microfluidic systems for oncotheranostics and other biomedical applications. Further improvement of such systems is necessary to more accurately model the tumor microenvironment, including the mechanical properties of tumor tissues, nutrient and oxygen concentration gradients, and imitation of dynamic interactions between tumor cells and healthy tissues. The creation of tumor-on-a-chip systems accurately mimicking the characteristics of individual patient tumors is the subject of research by many scientific groups and laboratories around the world. This may lead to significant progress in personalized medicine, providing the ability to select optimal dosages and combinations of drugs individually for a patient.

The microfluidic systems presented in this paper have several key advantages, making them a unique tool for research in the field of targeted delivery of nanoparticles. Firstly, they imitate the physiological conditions of tissues and vascular structures, which ensures reliable modeling of the processes of migration and accumulation of nanoparticles. Secondly, due to the use of porous media with an adjustable pore size, these systems allow varying experimental parameters and adapting them to different types of tissues. Finally, the technology of manufacturing microfluidic chips based on thermal compression and laser ablation ensures high accuracy and reproducibility at a relatively low cost, which makes them available for a wide range of studies. These advantages distinguish the systems presented from analogs and open new prospects for research in the field of nanomedicine and oncotheranostics.

## 5. Conclusions

The use of magnetic nanoparticles for the targeted delivery of drugs to tumor tissues is one of the promising areas in oncotheranostics. To achieve the advantages of this technique, it is important to precisely control the movement of nanoparticles using an external magnetic field, which allows them to be directed to specific areas of the tumor. Microfluidic systems developed for modeling tumor tissues (tumor-on-a-chip) are the perspective tool for studying the transport characteristics of magnetic nanoparticles.

In this work, models of tissues were prepared using planar sandwich-type polymer chips with basins into which polymer gels of various mean pore size and brain tissue of laboratory rats were deposited.

Two types of magnetic nanoparticles were prepared: magnetite nanoparticles (mMNPs) and carboxymethyl dextran coated SPIONS (CD-SPIONs).

Studies of the flow of an aqueous suspension of mMNPs in a capillary in the form of parallel flows with water showed a rapid agglomeration of mMNPs into microparticles within 10 min, then into large spatial structures causing the formation of laminar vortices within 20 min with subsequent destruction of the flow and, finally, the formation of a network covering all particles in the channel and consisting of magnetic fibers.

Studies of the magnetically driven migration of CD-SPIONs were carried out in polymer gel and brain tissue media. In polymer agar gels with mean pore sizes of 100–150 nm and 200–300 nm, no migration of CD-SPIONs was observed. In the gel with mean pore size of 350–400 nm, CD-SPIONs of 95–350 nm range were migrating in the field gradient of 40 T/m with differing velocities of 33, 16 and 5 μm/min correspondingly. No compression of the gel was observed. 

CD-SPIONs of 350 nm size did not penetrate the brain tissue and compressed it at a speed of 100 μm/min. Particles of 220 nm were also compressing the brain tissue, but at a considerably lower rate, and particles of 95 nm were partially penetrating the edge of the tissue and did not compress it. 

The agarose gels of sufficiently large mean pore size could be used in the tumor-on-a-chip format as a scaffold for tumor cells to which magnetic particles loaded with drugs could be operatively delivered in magnetic field for testing.

Further research could be aimed at improving the properties of nanoparticles to enhance their invasion into dense biological tissues as well as creating more complex tumor-on-a-chip models that consider not only the structural but also the biochemical properties of real tumors, making them more personalized.

## Figures and Tables

**Table 1:** Properties of polymer films used in formation of microfluidic chips.

Material Name	Thickness, mm	Density, g/cm^3^ at 20 °C	Melting Point, °C	MW, kDa	Structural Formula
Poly(methylmeth-acrylate) (PMMA) [39,40]	1.5	1.18	>100	100–150	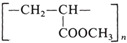
Polyethylene terephthalate (PET) [54,55,56]	0.5	1.38	>245	150	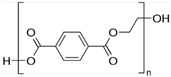
Polyethylene (PE) [57,58,59]	0.3	0.88–0.96	115–135	<200	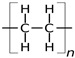
Polypropylene (PP) [60,61]	0.3	–	130–171	–	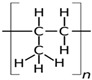
3M™ Scotch^®^ Trans-parent Film Tape 600 (polyvinylchloride (PVC) film/acrylic adhesive) [62]	0.52 0.38 (PVC base)	1.35–1.43	150–220	100–170	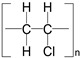

**Table 2:** Samples of magnetic nanoparticles: mMNPs and CD-SPIONs.

	Sample Name	Particle Size, nm	Particle Concentration, mg/mL
1	mMNP	46; 212	2
2	CD-SPION-1	95 ± 3	2.2–4.0
3	CD-SPION-2	220 ± 10	2.2–4.0
4	CD-SPION-3	350 ± 15	2.2–4.0
5	CD-SPION-4	820 ± 200	2.2–4.0

## Data Availability

The original contributions presented in this study are included in the article/Supplementary Material. Further inquiries can be directed to the corresponding authors.

## Supplementary Materials

The following supporting information can be downloaded at: https://www.mdpi.com/article/10.3390/nano14242030/s1.
