# Supplementary figures and images for: Magnetically Controlled Transport of Nanoparticles in Solid Tumor Tissues and Porous Media Using a Tumor-on-a-Chip Format
Source: Nanomaterials (Basel). 2024 Dec 17;14(24):2030. doi: 10.3390/nano14242030 (PMC11680056; doi:10.3390/nano14242030)

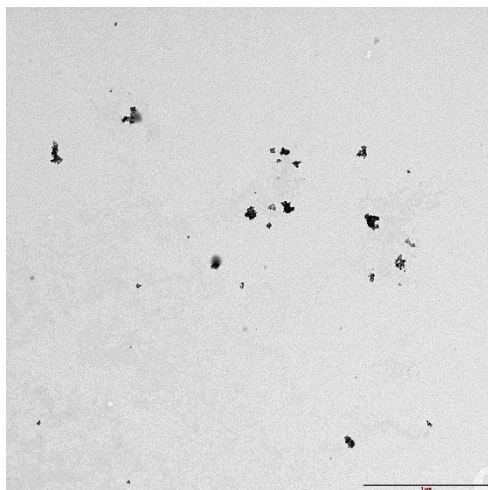

(a)

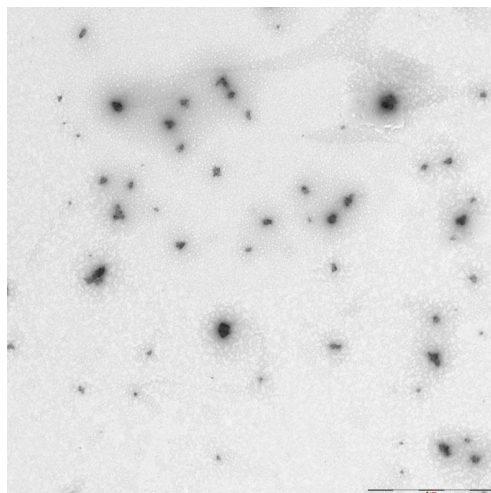

(b)

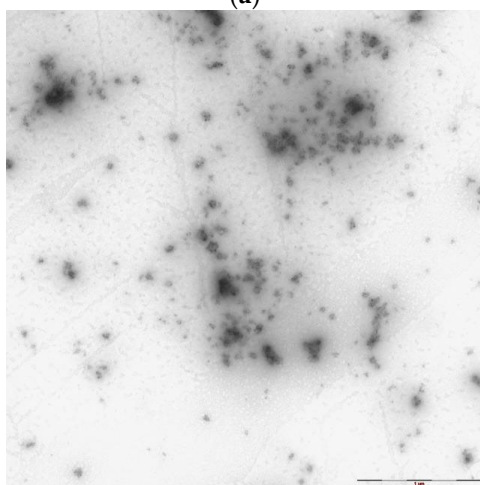

(c)

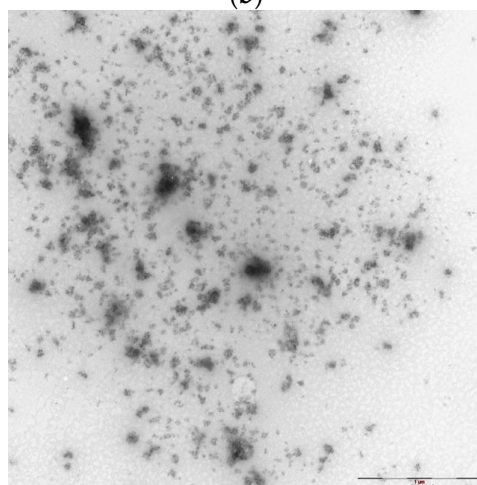

(d)

**Figure S1.** TEM images of SPIONs of different sizes: **(a)** 95 nm, **(b)** 220 nm, **(c)** 350 nm, **(d)** 820 nm.

Supplement: Supplementary file 1 [file nanomaterials-14-02030-s001.zip › nanomaterials-3312674-supplementary.pdf]
